# Morbidity and Mortality in Preterm Infants following Antacid Use: A Retrospective Audit

**DOI:** 10.1155/2016/9649162

**Published:** 2016-11-21

**Authors:** Natasha Singh, Aparna Dhayade, Abdel-Latif Mohamed, Tejasvi Vasant Chaudhari

**Affiliations:** ^1^Australian National University Medical School, Canberra, ACT 2601, Australia; ^2^Centenary Hospital for Women and Children, The Canberra Hospital, Woden, ACT 2606, Australia

## Abstract

*Background and Objectives*. Antacids are often prescribed to preterm infants due to misdiagnosis of gastro-oesophageal reflux. This suppresses gastric acidity, a major defence mechanism against infection. This study aims to determine if ranitidine and omeprazole use in very low birth weight (VLBW) neonates, <1500 grams, is associated with increased risk of late onset sepsis, necrotising enterocolitis (NEC), and mortality.* Methods*. Retrospective analysis was conducted on neonates, <1500 grams, born and admitted into the* Neonatal Intensive Care Unit* at* The Canberra Hospital* during the period from January 2008 to December 2012. Information regarding late onset sepsis, NEC, mortality, ranitidine/omeprazole use, and other neonatal/hospital factors was collected for each neonate.* Results*. 360 neonates were evaluated, 64 received ranitidine and/or omeprazole, and 296 had not. There were no statistically significant differences in incidence of late onset sepsis (OR = 0.52, CI = 0.24–1.1, and* p* = 0.117), NEC Stage 2 and above (OR = 0.4, CI = 0.05–3.2, and *p* = 0.7), or mortality (OR = 0.35, CI = 0.08–1.5, and *p* = 0.19) between the two groups. After adjusting significant differences in neonatal and hospital factors, risk of late onset sepsis was significantly lower in those that received ranitidine/omeprazole (OR = 0.28, CI = 0.13–0.65, and *p* = 0.003).* Conclusions*. Ranitidine and omeprazole use in VLBW preterm infants may not be associated with an increased risk of infection, NEC, and mortality.

## 1. Introduction

Refluxing of gastric contents is a common event in preterm infants and is often clinically misdiagnosed as gastro-oesophageal reflux disease (GORD) [1]. The diagnosis of GORD is a contentious issue due to lack of a truly valid diagnostic test in neonates and failure to fully identify the clinical syndrome [1, 2]. Clinical features of GORD include frequent vomiting, posits, effortless regurgitation of feeds, irritability, apnoea, and more disputative features of bradycardia and desaturations [1–5].

Antacids such as ranitidine and omeprazole are gastric acid secretion inhibitors and are commonly prescribed to preterm babies across neonatal intensive care units (NICUs) for management of reflux symptoms and GORD despite little evidence for efficacy and safety of their use in this age group [1, 3, 6, 7]. Prescription is in an off-labelled manner in preterm infants due to their perceived safety and possible benefits [3, 8–11]. Gastric juice acidity, however, provides a major non-immune defence barrier against infections in neonates [12]; hence the use of gastric inhibitors has been shown in various studies to assist onset of infections in preterm infants and children leading to morbidity and mortality [1, 12–15]. The pathophysiology behind this is still unfamiliar; however it has been proposed that blockade of acid secretion leads to gastrointestinal bacterial overgrowth prompting infections, as histamine-2 receptor (H2R) antagonists and proton pump inhibitors (PPIs) impede important leucocyte functions [13, 16–21]. Furthermore, recent evidence has found associations between the use of H2R antagonists and PPIs in very low birth weight (VLBW) neonates with an increased risk of necrotising enterocolitis (NEC) and mortality [6, 22–24]. NEC is a serious gastrointestinal emergency in preterm infants with the aetiology and pathogenesis being largely enigmatic [25].

The aim of this study was to compare rates of late onset sepsis, NEC, and mortality in preterm neonates <1500 grams in those who received ranitidine and/or omeprazole versus those who did not.

## 2. Methods

### 2.1. Study Population

VLBW preterm infants, <1500 grams, born and admitted to the NICU at* The Canberra Hospital* from January 2008 to December 2012, were included in the study. Patient data was obtained including gestational age, birth weight, APGAR scores, length of hospital stay, intrauterine growth restriction, use of ranitidine and/or omeprazole, and patent ductus arteriosus (PDA) using a neonatal database. Neonates with significant congenital abnormalities and immune deficiencies were excluded from the study. Neonates who developed infection or NEC within 48 hours of receiving ranitidine were not included in the treatment arm for analysis as it is highly unlikely that this was a result of the medication.

### 2.2. Outcome Measures

The primary outcome of this study was to compare the incidences of late onset sepsis, NEC, and mortality in premature infants exposed to ranitidine and/or omeprazole treatment. Late onset sepsis was defined as onset of sepsis 72 hours after birth and was diagnosed as presence of signs and symptoms of infection in conjunction with positive blood culture [26]. Data was also obtained for clinical/probable sepsis defined as presence of signs and symptoms of sepsis requiring treatment with antibiotics for ≥96 hours despite negative blood culture [27]. Incidence of pneumonia and urinary tract infections (UTI) was also evaluated. The presence of NEC and its Bell stage were determined based on standardised clinical or radiological criteria as described in patient notes [28]. Similarly, diagnosis of pneumonia and UTI was based on clinical/radiological criteria and positive urine culture with clinical signs of infection, respectively.

### 2.3. Data Collection

Lists of eligible neonates were obtained using the neonatal database at our institution. Discharge reports, paediatric medication charts, clinical notes, NICUS patient records, and pathology reports were used to obtain retrospective data. Data regarding neonatal and hospital factors, ranitidine and omeprazole use, and outcomes were collected for each neonate. Neonatal factors included gender, gestational age (GA), birth weight (BW), APGAR scores at 1 and 5 minutes, and presence and size of PDA. Hospital factors encompassed admission date, discharge date, length of hospital stay, use of central lines (umbilical venous catheter (UVC), umbilical arterial catheter (UAC), and percutaneously inserted central catheter (PICC)) and length of use, use of mechanical or invasive ventilation (conventional ventilation or high frequency oscillatory ventilation) and duration, use of continuous positive airway pressure (CPAP) and duration, type of feeding (expressed breast milk (EBM), formula, or both), and time to full feeds. If ranitidine or omeprazole was used, commencement age, duration, administration route, and indications for use (GORD, reflux, gastritis, and GIT bleeding) were documented. Clinical notes were also viewed to determine the symptoms and signs used for the diagnosis of GORD/reflux which included vomit, posits, apnoea, bradycardia, desaturations, aspiration, and/or irritability during feeds or immediately after feeds.

### 2.4. Statistics

All statistical analyses were conducted using IBM SPSS Statistics (SPSS for Windows, release 20.0.0. SPSS: an IBM company, Chicago, USA, 2012). Neonatal and hospital factors were summarised using frequencies and means by group defined by exposure to ranitidine/omeprazole. Statistical differences between groups were analysed via Pearson chi squared tests, Fisher's exact test, and independent sample *t*-tests.

Logistic regression was used to model the probability of late onset sepsis established on statistically important neonatal and hospital factors which could serve as confounders to the putative association between outcome and medication exposure. *p* values less than 0.05 were considered to be statistically significant.

### 2.5. Ethics

As this was a retrospective clinical audit, no consent procedures were required from individual patients. The study was approved by ACT Health Human Research Ethics Committee (ETHLR.13.128).

## 3. Results

### 3.1. Subject Disposition

**Figure 1:**
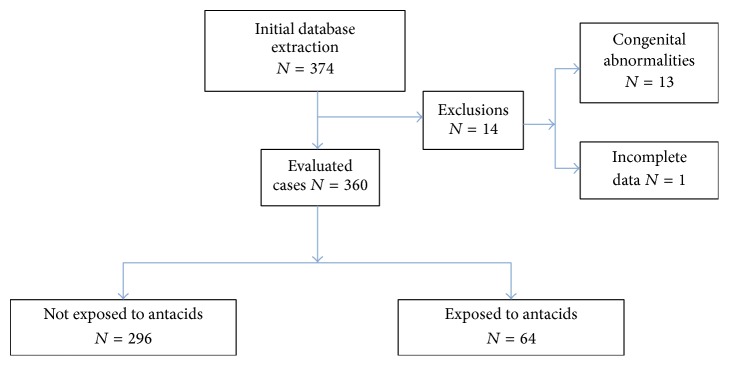
Flowchart of subject disposition.

A total of 360 neonates were evaluated. 13 neonates were excluded due to significant congenital abnormalities and 1 neonate did not have complete data records (Figure 1).

### 3.2. Ranitidine and Omeprazole Use

Of the 360 evaluated, 64 neonates received ranitidine (56 for symptoms of reflux and GORD, 4 for gastritis, and 4 for GIT bleeding) and 5 received omeprazole (2 indicated switch from ranitidine to omeprazole and 3 indicated symptoms of reflux/GORD). All 5 neonates that received omeprazole also received ranitidine. 296 neonates were not exposed to ranitidine and/or omeprazole. Fifty-five neonates (86%) received ranitidine enterally while 9 neonates (14%) received IV administration. All omeprazole doses were administered enterally. The mean dose for ranitidine was 6.9 ± 2.3 mg/Kg/day and for omeprazole 0.5 mg/Kg/day. The mean age of drug initiation was 37.5 ± 17.8 days for ranitidine and 72.4 ± 15 days for omeprazole. Ranitidine was used for an average of 25.6 ± 20.4 days while omeprazole was used on average for 30 ± 21 days.

### 3.3. Patient Clinical Characteristics 

Table 1 highlights the demographics and clinical characteristics of the included neonates. Gender and APGAR score at 5 minutes were comparable between the two groups. Neonates exposed to ranitidine/omeprazole were on average more premature compared with unexposed neonates (mean GA 27.3 weeks versus 28.6 weeks, *p* < 0.001). Exposed neonates were also on average smaller at birth (mean BW 1028.5 g versus 1127.7 g, *p* < 0.001) compared with unexposed neonates. Exposed neonates on average had a higher frequency of PDA ≥ 2 mm compared with unexposed neonates (25% versus 5.4%, *p* < 0.001).

Hospital factors (length of hospital stay, central line access, PICC access, ventilation, and feeding type) were also recorded for exposed and unexposed to ranitidine/omeprazole groups and are displayed in Table 2. Feeding type, umbilical line access, and mean duration of mechanical ventilation were similar between the exposed and unexposed groups. Neonates exposed to ranitidine/omeprazole had statistically significant higher frequencies of PICC access (70.3% versus 50.3%, *p* < 0.001), mechanical ventilation (79.7% versus 63.5%, *p* = 0.023), CPAP use (96.9% versus 83.8%, *p* = 0.014) and longer mean duration of hospital stay (74.7 days versus 42.1 days, *p* < 0.001), mean duration of PICC line (11.5 days versus 6.7 days, *p* = 0.01), and CPAP use (21.9 days versus 14.9 days, *p* = 0.013) than those not exposed to ranitidine/omeprazole.

### 3.4. Outcomes: Late Onset Sepsis, NEC, Mortality, Pneumonia, and UTI

Overall there were no statistically significant differences in the incidence of culture positive late onset sepsis (OR = 0.52, CI = 0.24–1.1, and *p* = 0.117), total late onset sepsis (OR = 0.73, CI = 0.4–1.34, and *p* = 0.384), NEC all stages (OR = 0.35, CI = 0.08–1.5, and *p* = 0.192), NEC Bell stage ≥2 (OR = 0.4, CI = 0.05–3.2, and *p* = 0.7), mortality (OR = 0.35, CI = 0.08–1.5, and *p* = 0.19), UTI (OR = 4.7, CI = 0.65–34.3, and *p* = 0.147), and pneumonia (OR = 1.9, CI = 0.36–9.9, and *p* = 0.613) between neonates exposed to ranitidine/omeprazole compared to neonates not exposed to the medications (Table 3). Due to low outcome magnitudes for NEC, mortality, UTI, and pneumonia in the exposed group further statistical analyses were not conducted.

Low GA, PICC line use, low APGAR score at 5 min, and length of hospital stay were all significant risk factors for culture positive late onset sepsis. These risk factors were included in a final bivariate logistic regression model for culture positive late onset sepsis (Table 4). After adjusting these risk factors, use of ranitidine/omeprazole appeared to lower the risk of total late onset sepsis (OR = 0.28, CI = 0.13–0.65, and *p* = 0.003). All above factors plus central line use were significant risk factors for total late onset sepsis (culture positive plus clinical late onset sepsis). After correcting for these factors using logistic regression, ranitidine/omeprazole use also appeared to lower the risk of total late onset sepsis.

## 4. Discussion

With increasing evidence of harmful outcomes associated with the use of H2R antagonists and PPIs in literature, the use of these medications was evaluated in a cohort of preterm neonates from our NICU. This retrospective analysis revealed that the use of ranitidine/omeprazole was not associated with the adverse outcomes of late onset sepsis, NEC, and mortality in VLBW neonates <1500 grams.

This is conflicting to previous reports. A prospective study by Terrin et al. [6] reported that ranitidine use in VLBW infants is associated with increased risk of infection, NEC, and mortality. This study, however, did not report on feeding type which is considered a potential independent risk factor for NEC [7]. This data was included in our study, with feeding types being comparable between exposed and non-exposed groups. Overall rate of culture positive sepsis and NEC Bell stage ≥2 was also greater in their study which may have influenced the incidence of study group outcomes. Although they reported “6 times” higher mortality in the ranitidine group compared to control group, the cause for deaths was not clarified and hence it may be difficult to attribute this to ranitidine use. Furthermore, a larger ranitidine dose was administered which may have led to a bigger impact, increasing the susceptibility of these adverse outcomes in naïve neonates. A retrospective case-control study conducted by Guillet et al. [23] also suggested an association between NEC and H2R antagonists; however feeding protocol, dosage, and duration of ranitidine use were again not reported. Overall rate of proven NEC was also higher than our study. Bianconi et al. [13] used a similar retrospective design and reported an association between ranitidine use and risk of late onset sepsis. Overall rates of culture positive sepsis were comparable to our study; however their duration of ranitidine use was longer which may have influenced the onset of sepsis. In addition, no ranitidine dosage was recorded. Finally Bilali et al. [22] reported an association between ranitidine use and outcomes of late onset sepsis and NEC after conducting a case-control study. Full demographic patient data was not reported, neither was overall rates of sepsis and NEC, ranitidine dosage, and duration. Furthermore, the association between NEC and ranitidine was not significant with 95% CI between 0.91 and 14.03. By addressing the factors of ranitidine dosage, age of administration and duration, and feeding type, our study provided a more accurate analysis of the association of these adverse outcomes with ranitidine. In addition, despite the fact that the medication group had more preterm and smaller neonates and had longer hospital stay and catheter days, the incidence of infection was found to be no different which further supports our findings. In fact, following correction of the confounding factors also related to late onset sepsis, we found that ranitidine/omeprazole use decreased the incidence of late onset sepsis. Although the incidence of UTI and pneumonia in the medication use group was higher, it was not statistically significant. Unfortunately, these values may be an underestimation (in both groups) due to underreporting and poor documentation in NICU notes regarding diagnosis of both these conditions.

One new aspect which was reported in our study was the age at which ranitidine was commenced. Since administration of ranitidine and omeprazole occurred on average 37 days and 72 days after birth, respectively, this may have allowed time for development and maturation of the immune system and other defences, reducing neonatal susceptibility to late onset sepsis or NEC. Due to a lack of information regarding this parameter in other studies, this potentially confounding association cannot be fully investigated. There may also be underreporting of studies which describe no association between ranitidine use and NEC and late onset sepsis.

Our study had several limitations. The retrospective design of this study limited our ability to control the assessment of exposures, outcomes, and other risk factors recorded for each neonate. The retrospective nature also inherently led to bias and difficulty when selecting a control group which in this case had significantly different risk factors to the exposed group but once controlled for did not impact results. Secondly, infants in the medication use group had much higher need for ventilation and PICC line and longer duration of PICC line and hospital stay. Although this could be explained by lower gestation and birth weight in the treatment group, ranitidine/omeprazole use may have had an independent effect on these morbidities which we cannot speculate. Our study did not compare late onset sepsis rates in controls after the mean age of commencement of ranitidine. This potentially means that rates of late onset sepsis were actually lower than reported in the control group as all late onset sepsis was included. Given that these findings are contradictory to current literature it is recommended that a randomised control trial be conducted to determine the underlying cause of this discrepancy and to establish a more definitive association between H2R antagonists and PPI with late onset sepsis, NEC, and mortality. Further research is also required in understanding the pathophysiology of NEC and development of immunity in neonates in order to fully understand the effect of medications in this vulnerable age group.

## 5. Conclusion

Ranitidine and omeprazole use in VLBW preterm neonates <1500 grams may not be associated with an increased risk of late onset sepsis, NEC, and mortality. Additional research in the form of a randomised control trial is required to explore this topic further and to investigate the underlying pathophysiology of NEC and late onset sepsis in preterm infants. Caution is still advised in the prescription of antacids in this highly susceptible age group due to limited information.

## Figures and Tables

**Table 1:** Neonatal factors in unexposed and exposed medication groups.

Neonate factor	Unexposed to ranitidine/omeprazole (*N* = 296)	Exposed to ranitidine/omeprazole (*N* = 64)	*p* value
Male gender, % (*n*)	51.7 (153)	50 (32)	0.915
Mean gestational age (weeks) + SD	28.6 ± 2.2	27.3 ± 1.6	<0.001
Mean birth weight (g) + SD	1127.7 ± 254	1028.5 ± 258.3	0.005
APGAR score <7 at 5 min, % (*n*)	26.8 (79)	23.4 (15)	0.693
PDA ≥ 2 mm, % (*n*)	5.4 (16)	25 (16)	<0.001

SD: standard deviation; PDA: patent ductus arteriosus.

**Table 2:** Hospital factors in unexposed and exposed medication groups.

Hospital factor	Unexposed to ranitidine/omeprazole (*N* = 296)	Exposed to ranitidine/omeprazole (*N* = 64)	*p* value
Mean length of hospital stay (days) + SD	42.1 ± 31	74.7 ± 50.5	<0.001
Umbilical line access, % (*n*)	83.4 (247)	93.8 (60)	0.055
PICC access, % (*n*)	50.3(149)	70.3 (45)	0.006
Mean duration of umbilical line (days) + SD	6.5 ± 5.1	8.1 ± 4.7	0.028
Mean duration of PICC line (days) + SD	6.7 ± 13.1	11.5 ±14.3	0.01
Mechanical ventilation, % (*n*)	63.5 (188)	79.7 (51)	<0.023
Mean duration of mechanical ventilation (days) + SD	5.1 ± 10.4	4.2 ± 11.3	0.563
CPAP, % (*n*)	83.8 (248)	96.9 (62)	0.014
Mean duration of CPAP (days) + SD	14.9 ± 21.1	21.9 ± 15	0.013
Feeding type, % (*n*)			
EBM	46 (136)	42.2 (27)	0.242
Formula	2.7 (8)	1.6 (1)	
Both	47 (139)	56.3 (36)	
Nil	4.4 (13)	0 (0)	
Time to full feeds (days) + SD	13.3 ± 9.6	15.4 ± 7.3	0.11

**Table 3:** Frequency and odds ratios of neonatal outcomes in group exposed to ranitidine/omeprazole.

Outcome	Not exposed to ranitidine/omeprazole (*N* = 296)	Exposed to ranitidine/omeprazole (*N* = 64)	Odds ratio (95% CI)	*p* value
Total late onset sepsis (culture positive + clinical sepsis)	33.1% (98)	26.6% (17)	0.73 (0.4–1.34)	0.384
Culture positive late onset sepsis	24% (71)	14.1% (9)	0.52 (0.24–1.1)	0.117
NEC (all stages)	8.4% (25)	3.1% (2)	0.35 (0.08–1.5)	0.192
NEC Bell stage ≥2	3.7% (11)	1.6% (1)	0.4 (0.05–3.2)	0.7
Mortality	8.4% (25)	3.1% (2)	0.35 (0.08–1.5)	0.19
UTI^†^	0.7% (2)	3.1% (2)	4.7 (0.65–34.3)	0.147
Pneumonia^†^	1.7% (5)	3.1% (2)	1.9 (0.36–9.9)	0.613

^†^Values may be underestimates due to underreporting and poor documentation in NICU.

**Table 4:** Bivariate logistic regression for culture positive late onset sepsis.

	Odds ratio (95% CI)	*p* value
Ranitidine/omeprazole use	0.28 (0.13–0.65)	0.003
Gestational age^†^		
≤25 weeks	5.3 (1.7–16.9)	0.005
26–30 weeks	1.8 (0.7–4.8)	0.21
Length of hospital stay^††^		
≥32 days	7.6 (0.9–66.1)	0.065
8–31 days	4.9 (0.55–44.4)	0.15
PICC access	3 (1.56–5.9)	0.001
APGAR 5 min score <7	1.53 (0.8–2.8)	0.18

^†^OR is relative to GA of 31–32 weeks.

^††^OR is relative to length of hospital stay ≤7 days.

## Abbreviations

BW: Birth weight
CPAP: Continuous positive airway pressure
CI: 95% Confidence interval
GA: Gestational age
GORD: Gastrooesophageal reflux disease
H2R: Histamine-2 receptor
HFOV: High frequency oscillatory ventilation
MV: Mechanical ventilation
NEC: Necrotising enterocolitis
NICU: Neonatal intensive care unit
OR: Odds ratio
PDA: Patent ductus arteriosus
PICC: Percutaneously inserted central catheter
UAC, UVC: Umbilical arterial catheter, umbilical venous catheter
UTI: Urinary tract infection
VLBW: Very low birth weight.

## References

[1] Birch J. L., Newell S. J. (2009). Gastrooesophageal reflux disease in preterm infants: current management and diagnostic dilemmas. *Archives of Disease in Childhood: Fetal and Neonatal Edition*.

[2] Dhillon A. S., Ewer A. K. (2004). Diagnosis and management of gastro-oesophageal reflux in preterm infants in neonatal intensive care units. *Acta Paediatrica*.

[3] Corvaglia L., Monari C., Martini S., Aceti A., Faldella G. (2013). Pharmacological therapy of gastroesophageal reflux in preterm infants. *Gastroenterology Research and Practice*.

[4] Wenzl T. G., Schenke S., Peschgens T., Silny J., Heimann G., Skopnik H. (2001). Association of apnea and nonacid gastroesophageal reflux in infants: investigations with the intraluminal impedance technique. *Pediatric Pulmonology*.

[5] Feranchak A. P., Orenstein S. R., Cohn J. F. (1994). Behaviors associated with onset of gastroesophageal reflux episodes in infants: prospective study using split-screen video and pH probe. *Clinical Pediatrics*.

[6] Terrin G., Passariello A., De Curtis M. (2012). Ranitidine is associated with infections, necrotizing enterocolitis, and fatal outcome in newborns. *Pediatrics*.

[7] Chandrasekaran M., Fleming P. (2014). Question 1: does the use of ranitidine increase the risk of NEC in preterm infants?. *Archives of Disease in Childhood*.

[8] Malcolm W. F., Gantz M., Martin R. J., Goldstein R. F., Goldberg R. N., Cotten C. M. (2008). Use of medications for gastroesophageal reflux at discharge among extremely low birth weight infants. *Pediatrics*.

[9] Cucchiara S., Minella R., Iervolino C. (1993). Omeprazole and high dose ranitidine in the treatment of refractory reflux oesophagitis. *Archives of Disease in Childhood*.

[10] Omari T. I., Haslam R. R., Lundborg P., Davidson G. P. (2007). Effect of omeprazole on acid gastroesophageal reflux and gastric acidity in preterm infants with pathological acid reflux. *Journal of Pediatric Gastroenterology and Nutrition*.

[11] Tighe M. P., Afzal N. A., Bevan A., Beattie R. M. (2009). Current pharmacological management of gastro-esophageal reflux in children: an evidence-based systematic review. *Pediatric Drugs*.

[12] Martinsen T. C., Bergh K., Waldum H. L. (2005). Gastric juice: a barrier against infectious diseases. *Basic and Clinical Pharmacology and Toxicology*.

[13] Bianconi S., Gudavalli M., Sutija V. G., Lopez A. L., Barillas-Arias L., Ron N. (2007). Ranitidine and late-onset sepsis in the neonatal intensive care unit. *Journal of Perinatal Medicine*.

[14] Beck-Sague C. M., Azimi P., Fonseca S. N. (1994). Bloodstream infections in neonatal intensive care unit patients: results of a multicenter study. *Pediatric Infectious Disease Journal*.

[15] Stoll B. J., Temprosa M., Tyson J. E. (1999). Dexamethasone therapy increases infection in very low birth weight infants. *Pediatrics*.

[16] Carrion V., Egan E. A. (1990). Prevention of neonatal necrotizing enterocolitis. *Journal of Pediatric Gastroenterology and Nutrition*.

[17] Lewis S. J., Franco S., Young G., O'Keefe S. J. D. (1996). Altered bowel function and duodenal bacterial overgrowth in patients treated with omeprazole. *Alimentary Pharmacology and Therapeutics*.

[18] Weber J. R., Angstwurm K., Rosenkranz T. (1997). Histamine (H1) receptor antagonist inhibits leukocyte rolling in pial vessels in the early phase of bacterial meningitis in rats. *Neuroscience Letters*.

[19] Yamaki K., Thorlacius H., Xie X., Lindbom L., Hedqvist P., Raud J. (1998). Characteristics of histamine-induced leukocyte rolling in the undisturbed microcirculation of the rat mesentery. *British Journal of Pharmacology*.

[20] Wandall J. H. (1992). Effects of omeprazole on neutrophil chemotaxis, super oxide production, degranulation, and translocation of cytochrome b-245. *Gut*.

[21] Agastaya G., West B. C., Callahan J. M. (2000). Omeprazole inhibits phagocytosis and acidification of phagolysosomes of normal human neutrophils in vitro. *Immunopharmacology and Immunotoxicology*.

[22] Bilali A., Galanis P., Bartsocas C., Sparos L., Velonakis E. (2013). H2-blocker therapy and incidence of necrotizing enterocolitis in preterm infants: a case-control study. *Pediatrics & Neonatology*.

[23] Guillet R., Stoll B. J., Cotten C. M. (2006). Association of H2-blocker therapy and higher incidence of necrotizing enterocolitis in very low birth weight infants. *Pediatrics*.

[24] More K., Athalye-Jape G., Rao S., Patole S. (2013). Association of inhibitors of gastric acid secretion and higher incidence of necrotizing enterocolitis in preterm very low-birth-weight infants. *American Journal of Perinatology*.

[25] Lin P. W., Stoll B. J. (2006). Necrotising enterocolitis. *The Lancet*.

[26] Klein J. O., Remington J. S., Klein J. O. (2001). Bacterial sepsis and meningitis. *Infectious Diseases of Fetus and Newborn Infant*.

[27] Lukacs S. L., Schrag S. J. (2012). Clinical sepsis in neonates and young infants, United States, 1988–2006. *Journal of Pediatrics*.

[28] Hack M., Horbar J. D., Malloy M. H., Tyson J. E., Wright E., Wright L. (1991). Very low birth weight outcomes of the National Institute of Child Health and Human Development Neonatal Network. *Pediatrics*.

